# Association between parental visitation and depressive symptoms among institutionalized children in Japan: a cross-sectional study

**DOI:** 10.1186/s12888-019-2111-x

**Published:** 2019-04-30

**Authors:** Aki Yazawa, Saeko Takada, Hanako Suzuki, Takashi X. Fujisawa, Akemi Tomoda

**Affiliations:** 1 Research Center for Child Mental Development, University of Fukui, 23-3 Matsuoka-Shimoaizuki, Eiheiji-cho, Fukui 910-1193 Japan; 2 Department of Comprehensive Psychology, Ritsumeikan University, 2-150 Iwakura-cho, Ibaraki, Osaka, Japan

**Keywords:** Child maltreatment, Institutionalized children, Depressive symptoms, Attachment, Parental visitation

## Abstract

**Background:**

Mental health problems are an important issue among institutionalized children. Although positive communication with parents is essential for children’s well-being, it has not been sufficiently verified how interactions with parents affect mental health among institutionalized children, who have experienced childhood adversity and likely lack secure attachment formation with their parents. The objectives of this study were to investigate the association between parental visitation and depressive symptoms among institutionalized children in Japan, and to explore whether the established security of attachment interacts with that association.

**Methods:**

A cross-sectional data from 399 institutionalized children aged 9 to 18 in Japan was used for the analysis. A mixed effects regression analysis was conducted to investigate the associations.

**Results:**

Institutionalized children who had parental visitation showed higher depressive symptoms than those who did not. In particular, father’s visitations were significantly associated with higher depressive symptoms. There was a significant interaction with score of secure attachment; children with low scores on secure attachment showed higher levels of depression with their father’s visitation, whereas children with high scores on secure attachment did not.

**Conclusions:**

Findings suggested that parental visitation and the frequency of visitation were not actually associated with better psychological status, but that instead, father’s visitations were associated with higher depressive symptoms among institutionalized children. It should be noted that our cross-sectional results cannot infer any causal relationship and do not emphasize that parental visitation should be avoided. However, it may be important to conduct careful assessment before starting parental visitation, especially when children seem to have problems with attachment formation.

## Background

Research has shown that mental health problems, such as social problems, internalizing and externalizing problems, and depression [1–6], are more frequently observed among children in out-of-home care, especially in child care institutions, than children in typical home care. This could be because most of them experienced stressful events in their original household, such as maltreatment, history of criminality by family members, parents’ mental disease, and low socioeconomic status, which are known risk factors for depression [7–9]. Institutionalization itself can also be a risk for depression as it is assumed to be a type of parental deprivation [10]. Additionally, the frequent turnover of primary caregivers due to the shift-work system in the institution has been shown to result in a lowered sense of belonging among these children [11], which can also induce depression [12]. Under the circumstances, it is considered an important task to reduce the psychological burden among institutionalized children.

In Japan, nearly 30,000 children were living in alternative care because their parents were either unable or unwilling to care for them properly as of 2015; nearly 60% of these children had a history of maltreatment [13], and over 80% of them were placed in institutions while the rest received care from foster parents, which is higher compared to other developed countries such as the U.S. (8%) [14, 15]. Since institutionalization is still common in the Japanese child welfare system, where nearly 50 children may live in the same facility [16], Japan has received recommendations to enhance alternative care, such as foster parents and family reunification, from the United Nations Committee on the Rights of the Child [17]. However, since there are not enough resources for child care institutions in Japan, in reality, it is crucial for ensuring children’s safety by providing a home-like, intimate environment at institutions with support from their own family members when possible, and to carefully assess and protect the connection between parents and children [18]. According to a report from the Ministry of Health, Labour and Welfare, nearly 50% of these children have some kind of contact (visitation, letters, or phone calls) with their parents [19].

In general, communication with parents is important for healthy development and well-being among children and adolescents [20, 21], although the results of previous studies on the impact of contacts with biological parents within children in foster care or institutions are controversial. Some studies have indicated that regular contact by biological parent(s) was positively associated with stronger attachment, children’s well-being, prevention of behavioral problems, and decreased depression scores [22–25]. On the other hand, a study revealed that one-third of children in foster care experienced conflicts when they have contacts with their biological parents [26], and workers in child care institutions or foster parents have shown reluctance to have visitation or regular contact with biological parents because it may cause psychological distress in children [27]. It was shown that 56% of adolescents in foster care recognized that contact with their parents was unhelpful for them [28], and children who established good relationships with both biological parents and foster parents were more likely to have loyalty conflicts [29].

Healthy communication with parents in early life could have fundamental and long-lasting impacts on the well-being through establishment of attachment. John Bowlby, the father of attachment theory, stated that infants have an ability to form an “affectional bond” with their caregiver (mostly the mother) to survive the world, as a primitive communication style [30]. Attachment experience with caregivers early in development is essential, as a child can internalize the attachment figure as a good one, and internalize the self-image as a valuable one to be treated in a good way through those experiences. This early attachment experience forms an “internal working model” which determines the form of interpersonal relationships throughout one’s life [31]. Moreover, established attachment affects the choice of health behaviors and hence both psychological and physical health in adulthood [32, 33]. A meta-analysis revealed that more than half of institutionalized children showed a disorganized pattern that cannot be classified in the three categories of attachment style (i.e., secure, avoidant, and ambivalent) [34], and it is estimated that 82% of maltreated children develop disorganized attachments [35]. The security of attachment may modify the quality of parent-child communications, especially among institutionalized children, who seem to lack secure attachment formation.

The objectives of this study were therefore (I) to investigate the association between parental visitation and depressive symptoms among institutionalized children in Japan and (II) to explore whether the established security of attachment interacts with that association.

## Methods

### Participants and data collection/procedures

The data used in this study were collected as part of a study on the Stress and Mental Health of Children in Residential Foster Care Facilities. Among 32 child care institutions asked to participate, 16 institutions across six prefectures in Japan agreed to participate in the survey. Children in fourth grade to sixth grade (age 9 to 12 years) completed the survey under supervision of facility staff members, who were able to explain the directions of questionnaires and answer questions if needed. Adolescents (age 12 to 18 years) were handed a packet of questionnaires, which they completed by themselves. A staff member for each child and adolescent completed a questionnaire about background information (e.g., maltreatment history, household situations) of the child and adolescent by referring to the record that was obtained as a part of the normal assessment process during the child’s institutionalization and preserved in each institution. After the questionnaires were completed, facility staff collected them and sent back to the researcher. Questionnaire data were obtained from 468 children and adolescents. Additional details of the survey profiles have been described elsewhere [5].

### Measures

#### Information on visitation

Total number of visits made in a year and relationship with the person who visited children were obtained. We categorized them as having visitation or not by (1) either father or mother, (2) father, (3) mother, and (4) grandparents, other relatives, and friends.

#### Depressive symptoms

The Japanese version of the Birleson Depression Self-Rating Scale for Children (DSRSC) was used for measurement of depressive symptoms. This scale consists of 18 items and was developed to evaluate depression in children [36]. The instrument is easy to use and has a predictive value (equal to or higher than 16) comparable with that of a psychiatric global rating of clinical range of depression obtained in an interview. The 18 items on the scale are rated with a 3-point Likert scale (range: 0–36). Cronbach’s alpha among the study participants was 0.62.

#### Attachment

The Internal Working Model Scale (IWMS) was used for the assessment of attachment security [37]. This questionnaire consists of 18 questions to assess three domains of attachment, namely, “secure,” “avoidant,” and “ambivalent.” The original version was developed by Hazan and Shaver [38] and the reliability and validity of the Japanese version has been confirmed [39]. As described in the Introduction, given that most institutionalized children seem to have established a disorganized attachment pattern that cannot be classified in the above-mentioned three categories, we used the secure attachment score of IWMS alone to focus on the variation of the insecurity among the participants. Cronbach’s alpha among the present study participants was 0.81.

#### Demographics, history of maltreatment, and social support

Children’s basic information included their age (in years), gender, family composition, and whether children were exposed to any kinds of child maltreatment such as physical abuse, sexual abuse, emotional abuse, neglect, and/or domestic violence. Social support, which is described as psychological and material resources exchanged between individual network members to promote personal well-being [40], was assessed with the Perceived Emotional Support Scale; the scale was originally developed by Sarason et al. [41] and then modified by Hisada et al. [42]. Since the original version was established for assessing social support in youth, the wording was slightly changed for younger children. It consists of 16 questions, such as “when you feel depressed, the person cheers you up,” or “when you fail to do something, the person will help you,” to assess the perception of emotional support from close others. Each item was rated with a 4-point Likert scale for each person. The Cronbach’s alpha was 0.99, 0.99, and 0.96 for mother, father, and care workers among this study’s participants, respectively.

### Data analysis

After excluding people who did not provide data on history of maltreatment, depression score, age, and gender, the size of the final sample used in the analysis was 399. Among them, 357 participants completed the questionnaire on attachment security. Since it was presupposed that depression symptoms among children may differ by household type (i.e., has both parents, single mother, single father, or has no parent) with which main analysis (i.e., visitation x depression) was conducted, DSRSC scores were compared between each group before the main analyses, controlling for age, gender, frequency of maltreatment (0, 1, 2 or more times), and perceived social support from care workers (ranges 16–80, which was then categorized into tertiles) using a mixed effects regression analysis with a random effects model to account for multiple individuals in each institution.

A mixed effects regression analysis was also used to investigate the association between visitation and DSRSC score. The outcome was used as a continuous variable as it was distributed normally. An association between mother’s or father’s visitation and DSRSC score was only investigated among children who have a mother or father in their household, respectively. Covariates included age, gender, frequency of maltreatment, and perceived social support from mother or father (if the child has a mother or father in their household) and care workers. Missing information for the covariates was included as missing categories in order to maintain the statistical power. Parental visitation by either mother or father, mother’s visitation, father’s visitation, visitation by grandparents, other relatives, and friends, and the total number of visits made in a year (none; 1 to 6 times a year; 7 or more times a year) were included in separate models. Each model included respective visiting persons regardless of other visiting persons. Standardized score of secure attachment and the interaction term with parental visitation were then included in the analysis to examine the potential moderating effect on the association between parental visitation and the DSRSC score.

All statistical analyses were conducted using Stata 13.1 (College Station, TX, USA). The level of statistical significance was set at *p* < 0.05 and *p* < 0.10 for the interaction effect (two-tailed).

## Results

### Characteristics of the study participants

Table 1 shows the descriptive statistics. Among the 399 participants included in the analysis, the mean age was 13.3 and females comprised 52.9% of the participants. The mean score of DSRSC was 14.6 (SD 6.3) and 43.4% of them showed depressive state (i.e., having a DSRSC score equal to or higher than 16). Among the participants, 41.6% had mother’s visitation and 28.1% had father’s visitation, while 26.6% of them did not have any visitation in a year.

**Table 1:** Characteristics of the participants (*N* = 399)

	Mean [SD] / n (%)
Age	13.3 [2.4]
Gender (% Female)	211 (52.9)
DSRSC score	14.6 [6.3]
Household type	
Having both parents	128 (32.1)
Single mother	136 (34.1)
Single father	61 (15.3)
Do not have a parent (orphans)	28 (7.0)
Visitation	
Either mother or father ^a^	183 (49.3)
Mother ^a^	129 (41.6)
Father ^a^	66 (28.1)
Grandparents, other relatives, and friends	46 (11.5)
Number of visits (per year)	
No visit	106 (26.6)
1 to 6 times	105 (26.3)
6 or more times	137 (34.3)
Experience of maltreatment	
Physical abuse	97 (24.3)
Emotional abuse	102 (25.6)
Sexual abuse	20 (5.0)
Neglect	158 (39.6)
Domestic violence	65 (16.3)
Frequency of maltreatment	
None	123 (30.8)
1	118 (29.6)
2	66 (16.5)
3 or more	56 (14.0)
Perceived social support	
Mother	
Low	91 (22.8)
Middle	102 (25.6)
High	92 (23.1)
Father	
Low	93 (25.2)
Middle	91 (24.7)
High	94 (25.5)
Care workers	
Low	102 (25.6)
Middle	116 (29.1)
High	112 (28.1)
Secure scores of Internal Working Model Scale	19.9 [6.3]

Mean and standard deviation for continuous variables and number of participants and the percentage for categorical variables are shown (mean [SD]/n (%)). The figures do not sum to 100% in the section as data on missing values are not shown

^a^Only includes biological parents

Depression score was highest among children who have both parents and was lowest among children having no parents (Mean [SD] was 15.9 [6.5] vs. 13.0 [6.0], respectively; data not shown in Tables). After controlling for covariates, a significant difference was still observed (coefficient = − 2.8, 95% confidence interval [CI] = − 5.2, − 0.42; data not shown in Tables).

### Types of visitor, number of visits, and depressive symptoms

Table 2 shows the association between visitation and the DSRSC score. Visitation by either mother or father was positively associated with DSRSC score (coefficient = 2.9, 95% CI = 0.80, 5.0, Model 1). When separately analyzed, mother’s visitation was not significantly associated with DSRSC score (Model 2) while father’s visitation was positively associated with DSRSC score (coefficient = 2.3, 95% CI = 0.51, 4.0, Model 3). Although there was a marginal significance (*p* = 0.080), visitation by grandparents, other relatives, and friends was inversely associated with the DSRSC score (coefficient = − 1.60, 95% CI = − 3.4, 0.19, Model 4). Total number of visits in the past year was not associated with DSRSC score.

**Table 2:** Association between visitation and depression score

	Model 1 (*n* = 123)	Model 2 (*n* = 237)	Model 3 (*n* = 189)	Model 4 (*n* = 399)	Model 5 (*n* = 348)
Visitation ^a^					
Either mother or father ^b^	2.9** [0.80, 5.0]				
Mother ^b^		−0.62 [− 2.2, 0.92]			
Father ^b^			2.3* [0.51, 4.0]		
Grandparents, other relatives, and friends				−1.60^†^ [−3.4, 0.19]	
Number of visits (ref. None) (per year)					
1 to 6 times					0.11 [−1.5, 1.7]
6 or more times					0.71 [−0.81, 2.2]
Age	0.67* [0.14, 1.2]	0.35^†^ [−0.0035, 0.69]	0.56** [0.16, 0.95]	0.34* [0.078, 0.59]	0.36* [0.078, 0.64]
Gender (ref. Female)	−4.0** [−6.1, −1.9]	−3.0** [−4.6, − 1.5]	−3.1** [− 4.7, − 1.4]	−3.2* [− 4.3, − 2.0]	− 3.4** [− 4.6, − 2.1]
Frequency of maltreatment (ref. None)					
1	0.69 [− 2.1, 3.5]	0.58 [− 1.4, 2.5]	0.32 [− 1.9, 2.5]	−0.098 [− 1.6, 1.4]	− 0.11 [− 1.6, 1.4]
2	2.4 [−0.66, 5.5]	0.94 [−1.4, 3.2]	0.81 [−1.5, 3.2]	0.038 [−1.7, 1.8]	−0.15 [−2.0, 1.7]
3 or more	1.8 [−1.3, 4.9]	2.6* [0.31, 4.8]	1.9 [−0.68, 4.4]	2.2* [0.36, 4.1]	2.3* [0.29, 4.2]
Social support from care workers (ref. Low)					
Middle	−0.010 [−2.9, 2.9]	−0.73 [− 2.8, 1.4]	0.052 [− 2.3, 2.4]	−1.6* [3.1, − 0.030]	− 1.7* [−3.3, − 0.040]
High	−0.94 [−4.1, 2.2]	−2.3^†^ [−4.7, 0.043]	−1.1^†^[−3.6, 1.3]	−3.5* [−5.1, −1.8]	−3.4** [−5.1, −1.6]
Social support from mother (ref. Low)					
Middle	0.38 [−2.9, 3.7]	−1.7 [−4.0, 0.53]			
High	1.3 [−2.8, 5.4]	−0.90 [−3.3, 1.5]			
Social support from father (ref. Low)					
Middle	−4.1* [−7.3, −0.85]		−3.1* [−5.5, − 0.59]		
High	−3.9 [−7.6, −0.16]		−2.8* [−5.5, −0.18]		

Coefficient and 95% confidence interval are shown. *: *p* < 0.05; ^†^: *p* < 0.1

A mixed effects regression analysis with s random effects model was used to account for multiple individuals in each institution. Missing values are not shown in the table

Each model was conducted among participants who have parent(s) to test the impact of visitation

^a^Each model included the respective visiting persons regardless of other visiting persons

^b^Only includes biological parents

Although it was not the main hypothesis in this study, given that household type (i.e., has both parents, single mother, single father, or has no parent) was a significant determinant of depressive symptoms, we additionally investigated the association by taking into account the interaction of household type on the association. Results showed that there was no significant interaction between having only father and father’s visitation, while there was a significant interaction between having only mother and mother’s visitation (coefficient for the interaction term = − 3.2, *p* = 0.041; Appendix); children of single mothers had lower DSRSC scores when having mother’s visitation (*p* = 0.015), while children who have both parents did not.

### Interaction of attachment security on the association between visitation and depressive symptoms

**Fig. 1:**
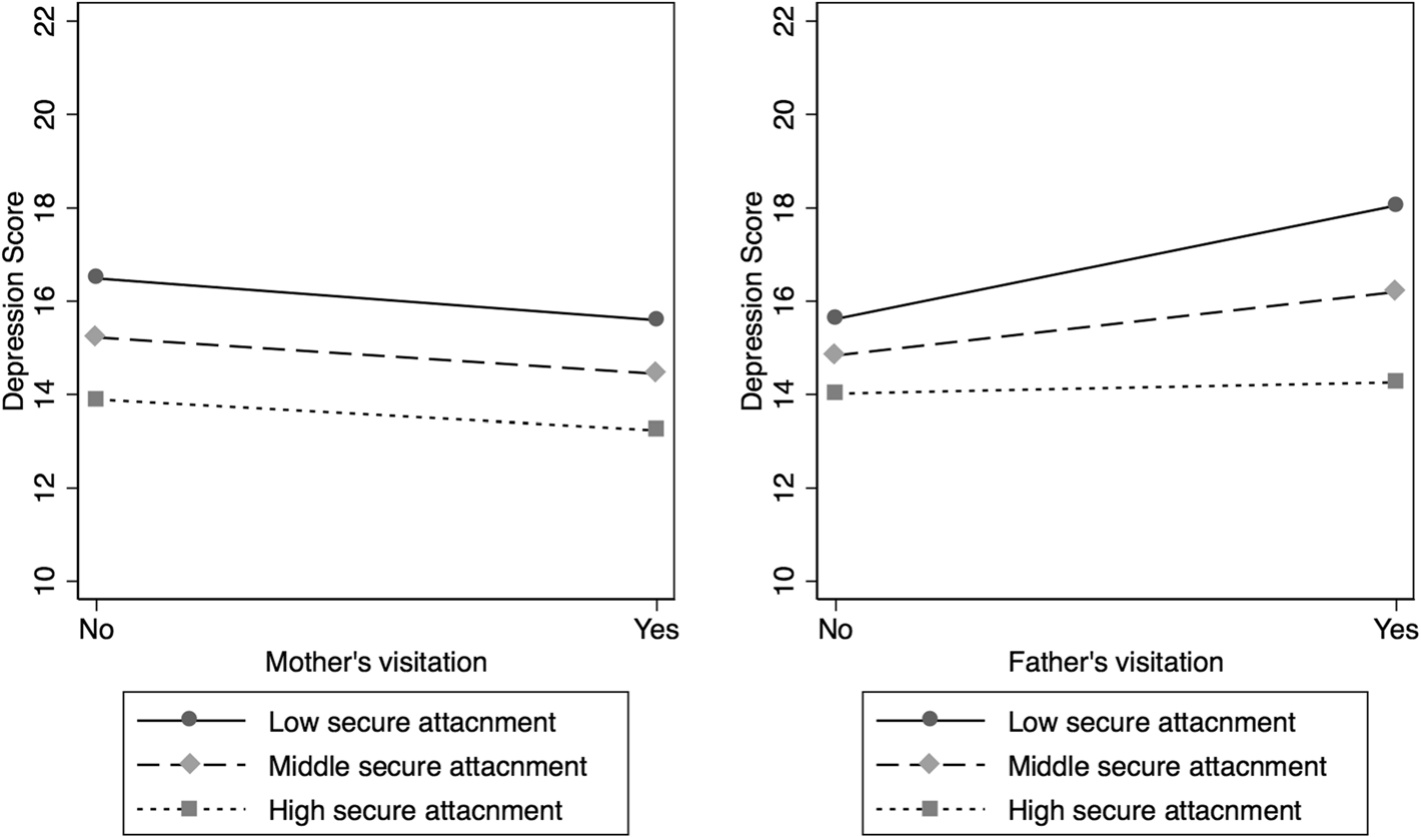
Interaction between parental visitation and secure attachment on depression score

The IWMS secure attachment score was significantly inversely associated with DSRSC score (coefficient = − 1.9, 95% CI = − 2.5, − 1.2; Model 1 in Table 3). The interaction between the secure attachment score and father’s visitation was also significant (coefficient = − 1.8, 95% CI = − 3.6, 0.045, Model 3); children who have low secure attachment scores (the lowest tertile among the participants) showed higher levels of DSRSC score when having father’s visitation compared to when having no father’s visitation (*p* = 0.012), while children having high secure attachment scores did not show such a difference (Fig. 1). There was no significant interaction between the level of secure attachment and mother’s visitation (Model 5 in Table 3; Fig. 1).

**Table 3:** Association between secure attachment score, father’s visitation and depression score

	Model 1 (*n* = 331)	Model 2 (*n* = 171)	Model 3 (*n* = 171)	Model 4 (*n* = 215)	Model 5 (*n* = 215)
Secure attachment score	−1.9** [−2.5, − 1.2]	− 2.0** [− 2.9, − 1.1]	− 1.2^†^ [− 2.3, 0.022]	−2.0** [− 2.8, − 1.2]	−2.0** [− 3.2, − 0.85]
Father’s visitation ^a^		1.5^†^ [− 0.27, 3.3]	1.3 [− 0.47, 3.1]		
Secure attachment score x Father’s visitation			−1.8^†^ [−3.6, 0.045]		
Mother’s visitation ^a^				−0.73 [−2.3, 0.84]	−0.78 [−2.3, 0.78]
Secure attachment score x Mother’s visitation					0.19 [−1.39, 1.77]

Coefficient and 95% confidence interval are shown. *: *p* < 0.05; ^†^: *p* < 0.1

A mixed effects regression analysis with s random effects model was used to account for multiple individuals in each institution controlling for age, gender, frequency of maltreatment, and social support from care workers. Models 2 and 3 additionally controlled for social support from the father, while Models 4 and 5 controlled for social support from mother

Each model was conducted among participants who have parent(s) to test the impact of visitation

^a^Only includes biological parents. Each model included the respective visiting persons regardless of other visiting persons

## Discussion

### Summary of the findings

This is the first study to examine the association between parental visitation and depressive symptoms among institutionalized children in Japan. In the present study, we found that father’s visitation was significantly associated with higher depressive symptoms. Secure attachment score was inversely associated with depression score and there was a significant interaction between secure attachment and father’s visitation; children with low secure attachment scores showed higher depression scores when having father’s visitation whereas children with high attachment scores did not.

### Household type and depression score

Surprisingly, depression score was highest among children who have both parents and was lowest among orphans. The situation in which they needed to enter an institution even though they have both parents may indicate that the circumstances in their original household (e.g., maltreatment) were more serious than those of children who have one or no parent, so they showed higher depression scores. Among this study’s participants, the frequency of maltreatment children experienced was greater among children who have both parents than orphans (Mean [SD] = 1.5 [1.2] vs. 0.61 [0.96], respectively; data not shown in Tables). Previous studies have shown that maltreated children can easily feel self-blame and responsibility for the institutionalization [43]; for example, Quas et al. [44] have shown that abuse severity was positively associated with self-blame among sexually abused children, which could be a psychological burden for these children. There is also a study pointing out the possibility that inconsistent contact with parents can be more stressful than having no contact, as it can cause ambiguous loss which disturbs the grieving process from moving forward [25].

### Association between visitation and depression score

A positive association was observed between father’s visitation and depression score, while there was no significant association between mother’s visitation and depression score. Although it is considered that visitation can be the primary intervention for maintaining parent-child relationships [18], our results showed a higher psychological burden among children having father’s visitation. Given that Moyers et al. [45] have found that 63% of children who took foster placements had contact with someone who was detrimental to them (e.g., a perpetrator of abuse) after 1 year of the placement, and they were more likely to have difficulty in expressing their emotion, a relationship with parents could affect the association and be reflected in a heightened depression score. Although data on the perpetrator of abuse was available only for 223 children, we certificated that the association was still significant when we exclude children who had been abused by their father (coefficient = 2.4, 95% CI = 0.23, 4.7, *p* = 0.031; data not shown in Tables). Given that many of the children had experienced a complex history of maltreatment (e.g., the dual burden of neglect from the mother and physical abuse from the father), future study should address this point (i.e., impact of visitation by perpetrator) using data with much bigger and detailed sample. As another possible situation, although there was no interaction between father’s visitation and household type (i.e., single-father household vs. household with both parents) (Appendix), it is possible that children who have frequent father’s visitation tend to experience less support from their mother and reflected in higher depressive symptoms. Since the total number of visits was not associated with depression score, considerable attention should be paid to the quality of each visitation.

Although it was of borderline significance, visitation by grandparents, other relatives, and friends was associated with lowered depression score. This is consistent with the finding by Simsek et al. [24] that children having regular contact with their parents or relatives showed fewer internalizing problems, although they did not analyze the association separately for contacts with parents and relatives. This finding may indicate that child welfare should concern the involvement not only of parents but also of extended family members and friends (i.e., any people who care for the children regardless of the blood relationship), so that children would feel supported. Future study should focus not only on parents but also on other close adults.

For children who only have a mother, mother’s visitation was protective against their depressive symptoms (Appendix). This is consistent with a previous study that showed that frequent contact with the biological mother was associated with lower levels of depression, although this was a marginal association among children in foster care [25]. The present data showed that frequency of maltreatment was lower among single-mother households than households with both parents (Mean [SD] = 1.5 [1.2] vs. 1.2 [1.1], respectively; data not shown in Tables). Considering that single-mother households are more likely to have economic disadvantages in Japan [46], it may reflect the result of reluctantly-decided institutionalization in single-mother households. This speculation needs to be tested with more detailed data.

### Interaction effect of attachment security

As for the association with attachment, there was a significant interaction between the secure attachment score and father’s visitation on depressive symptoms; children who have low secure attachment scores showed higher levels of depression when having father’s visitation, whereas those with high scores did not (Fig. 1). This is in line with a previous study, which showed that children with stronger attachment were less likely to take psychiatric medication among children in foster care [22]. They also reported that frequent contact with mother was associated with stronger attachment to their mother, although they targeted families in which reunification is a goal. Given that secure attachment relationships enhanced the child’s coping capacities toward stress [47], children with higher secure attachment would have a more stable mental status even if they regard visitation as a stressor.

The authors expected that children who have a high secure attachment would show decreased depression scores when having father’s visitation. However, it should be noted that the depression score remained at the same level when having father’s visitation among children with high secure attachment compared to children who have no visitation. Given that 54% of institutionalized children and 82% of children who have experienced maltreatment are supposed to have a disorganized style of attachment as stated in the Introduction [34, 35], children who had a higher secure attachment score in this study (i.e., the highest tertile among the participants) did not seem to have formed a secure attachment, which is comparable to that of healthy population.

### Strengths and limitations

This study extends earlier research on mental health among institutionalized children in several ways. One of the strengths of the study is the sample size. Compared to previous studies conducted with institutionalized children, which had sample sizes that were relatively small of around 100–200 [1–4], this study collected a larger sample size. Another strength was that this study was conducted on a multisite basis, from rural to urban areas across Japan.

The current study has several important limitations. First, the cross-sectional design of the study prevented us from making causal inferences. Second, although many variables were included as covariates, we did not have data on several variables that could have better explained the studied association, such as the diverse history of adverse experiences that each child has. As for the type and perpetrator of maltreatment, we did not have a large enough sample size for stratifying the analysis (e.g., only 20 had experienced sexual abuse), and many of the children had experienced a complex history of maltreatment, which may have different impacts on depression among children. Third, we used the IWMS in this study to assess attachment style; observational or representational procedures are desirable for assessing childhood attachment. Fourth, since this is a retrospective study, we only analyzed the number of visitations. Future studies should examine not only the quantity but also the quality of visitations and how children perceived their parental visitation. For example, in the U.S., there are organizations to monitor and assess the quality of the visitation before reunification (i.e., supervised visitation), and a study showed that children who had more consistent and frequent visitation showed better mental health status [22].

## Conclusions

The number of children who have been institutionalized because of child maltreatment or other adverse experiences keeps increasing in Japan, where child care institutions do not have adequate resources. Yamamoto et al. [48] reported in 2010 that more than half of institutionalized children returned home without resolving their household problems, and 11–14% of those children were institutionalized again within 1 year. Under these circumstances, the relationship between biological parents and children is regarded as inseparable and it is thus an urgent task to develop an assessment system for evaluating the parent-child relationship.

The present study indicated that the association between visitation and psychological well-being among children depends on each child’s situation (e.g., either having father/mother or not) and interacted with the established attachment security. It is the first study that indicated a positive association between parental visitation and depressive symptoms; it is significant as it is widely believed that parental visitation is important for maintaining parent-child relationships [18]. It may be important to conduct precise assessment before starting parental visitation, especially when children seem to have problems with attachment. It should be noted that the present study does not emphasize that parental visitation should be avoided. Professionals should pay attention to the attachment figure of each child and their perception of the relationship with their parents regardless of the presence or frequency of visitation.

## Figures and Tables

**Fig. 2:**
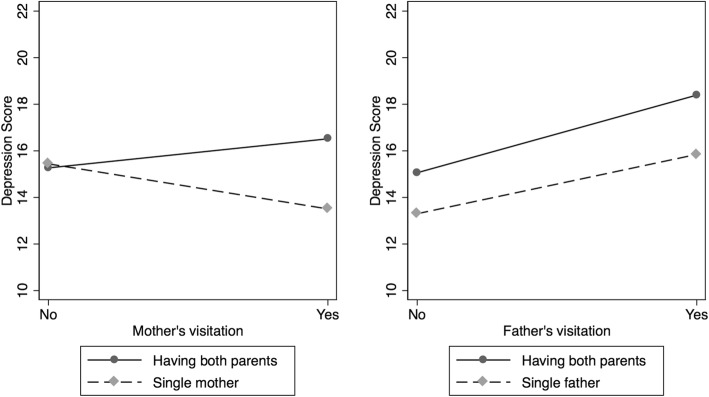
Interaction between parental visitation and parenting style on depression score

## Abbreviations

DSRSC: The Birleson Depression Self-Rating Scale for Children
IWMS: The Internal Working Model Scale

## References

[1] Padmaja G, Sushma B, Agarwal S (2014). Psychosocial problems and well-being in institutionalized and non-institutionalized children. J Humanit Soc Sci.

[2] Palacios J, Moreno C, Roman M (2013). Social competence in internationally adopted and institutionalized children. Early Child Res Q.

[3] Dell'Aglio DD, Hutz CS (2004). Depression and school achievement of institutionalized children and adolescents. Psicologia: Reflexão e Crítica.

[4] Wathier JL, Dell'Aglio DD (2007). Depressive symptoms and stressful events in children and adolescents in the institutionalized context. Revista de Psiquiatria do Rio Grande do Sul.

[5] Suzuki H, Tomoda A (2015). Roles of attachment and self-esteem: impact of early life stress on depressive symptoms among Japanese institutionalized children. BMC Psychiatry.

[6] Boss K, Zeanah CH, Fox NA, Drury SS, McLaughlin KA, Nelson CA (2011). Psychiatric outcomes in young children with a history of institutionalization. Harvard Rev Psychiatry.

[7] Sternberg KJ, Lamb ME, Gutterman E, Abbott CB (2006). Effects of early and later family violence on children's behavior problems and depression: a longitudinal, multi-informant perspective. Child Abuse Negl.

[8] Masten AS, Garmezy N. Risk, vulnerability and protective factors in developmental psychopathology. Adv Clin Child Psychol. 1985;8:1–52. Nova York: Plenum.

[9] Heim C, Nemeroff CB (2001). The role of childhood trauma in the neurobiology of mood and anxiety disorders: preclinical and clinical studies. Biol Psychiatry.

[10] Pollak SD, Nelson C, Schlaak MF, Roeber BJ, Wewerka SS, Wiik KL (2010). Neurodevelopmental effects of early deprivation in post-institutionalized children. Child Dev.

[11] Barone L, Dellagiulia A, Lionetti F (2015). When the primary caregiver is missing: investigating proximal and distal variables involved in institutionalized children's adjustment. Child Abuse Rev.

[12] Hagerty BM, Williams RA (1999). The effects of sense of belonging, social support, conflict, and loneliness on depression. Nurs Res.

[13] Survey on institutionalized children in Japan (in Japanese) (2015). Ministry of Health, Labour and welfare.

[14] Takahashi M, Tokunaga S, Takahashi E, Ieko N, Matsuoka N, Kondo M. Systematic review of the outcome of social nursing in Japan (in Japanese). Nippon Foundation. 2017. https://www.nippon-foundation.or.jp/app/uploads/2018/12/wha_pro_hap_10.pdf. Accessed 23 Apr 2019.

[15] Child Welfare Information Gateway (2017). Foster Care Statistics 2015.

[16] Wada I, Igarashi A (2014). The social cost of childcare in Japan. Child Youth Serv Rev.

[17] Japan (2014). Children in institutions denied family life. Human Rights Watch.

[18] Mallon G, Leashore B (2002). Preface. Child Welfare.

[19] Survey on Children in Children’s Nursing Homes (in Japanese) (2015). Ministry of Health, Labour and Welfare, Equal Employment, Children and Families Bureau.

[20] Branje SJT, Hale WW, Frijns T, Meeus WHJ (2010). Longitudinal associations between perceived parent-child relationship quality and depressive symptoms in adolescence. J Abnorm Child Psychol.

[21] Xiao Z, Li X, Stanton B (2011). Perceptions of parent-adolescent communication within families: it is a matter of perspective. Psychol Health Med.

[22] McWey LM, Mullis AK (2004). Improving the lives of children in foster care: the impact of supervised visitation. Fam Relat.

[23] Cantos AL, Gries LT, Slis V (1997). Behavioral correlates of parental visiting during family foster care. Child Welfare..

[24] Simsek Z, Erol N, Oztop D, Munir K (2007). Prevalence and predictors of emotional and behavioral problems reported by teachers among institutionally reared children and adolescents in Turkish orphanages compared with community controls. Child Youth Serv Rev.

[25] McWey LM, Acock A, Porter B (2010). The impact of continued contact with biological parents upon the mental health of children in foster care. Child Youth Serv Rev.

[26] Neil E, Beek M, Schofield G (2003). Thinking about and managing contact in permanent placements: the differences and similarities between adoptive parents and foster carers. Clin Child Psychol Psychiatry.

[27] Mennen FE, O’Keefe M (2005). Informed decisions in child welfare: the use of attachment theory. Child Youth Serv Rev.

[28] Farmer E, Pollock S (1998). Sexually abused and abusing children in substitute care.

[29] Leathers SJ (2003). Parental visiting, conflicting allegiances, and emotional and behavioral problems among foster children. Fam Relat.

[30] Bowlby J (1979). The making and breaking of affectional bonds.

[31] Toda K (1988). Interpersonal relationships and attachment styles in late adolescence: consideration from working models (in Japanese). The Japanese Association of Educational Psychology.

[32] Huntsinger ET, Luecken LJ (2004). Attachment relationships and health behavior: the mediational role of self-esteem. Psychol Health.

[33] Puig J, Englund MM, Simpson JA, Collins WA (2013). Predicting adult physical illness from infant attachment: a prospective longitudinal study. Health Psychol.

[34] Lionetti F, Pastore M, Barone L (2015). Attachment in institutionalized children: a review and meta-analysis. Child Abuse Negl.

[35] Carlson V, Cicchetti D, Barnett D, Braunwald KG (1989). Finding order in disorganization: lessons from research on maltreated infants’ attachments to their caregivers. Child maltreatment: theory and research on the causes and consequences of child abuse and neglect.

[36] Birleson P, Hudson I, Buchanan DG, Wolff S (1987). Clinical evaluation of a self-rating scale for depressive disorder in childhood (depression self-rating scale). J Child Psychol Psychiatry.

[37] Toda K (1988). Basic interpersonal relationships and attachment style in the late adolescence-examination from working models.

[38] Hazan C, Shaver P (1987). Romantic love conceptualized as an attachment process. J Pers Soc Psychol.

[39] Matsumoto T (1998). Reliability of the internal working models: among late adolescent males (in Japanese). Akita Ronso.

[40] Tanaka K, Social support (1997). The Japanese Association of Health Psychology Ed (in Japanese). Dictionary of Health Psychology. Jitsumukyoiku-shuppan.

[41] Sarason BR, Shearin EN, Pierce GR, Sarason IG (1987). Interrelations of social support measures: theoretical and practical implications. J Pers Soc Psychol.

[42] Hisada M, Senda S, Minoguchi M (1989). Developing a social support scale for students (1). The 30th annual meeting of the Japanese Society of Social Psychology.

[43] Herman JL (1997). Trauma and recovery.

[44] Quas JA, Goodman GS, Jones D (2003). Predictors of attributions of self-blame and internalizing behavior problems in sexually abused children. J Child Psychol Psychiatry.

[45] Moyers S, Farmer E, Lipscombe J (2006). Contact with family members and its impact on adolescents and their foster placements. Br J Soc Work.

[46] Raymo JM, Zhou Y (2012). Living arrangements and the well-being of single mothers in Japan. Popul Res Policy Rev.

[47] Schore AN (2001). Effects of a secure attachment relationship on right brain development, affect regulation, and infant mental health. Infant Ment Health J.

[48] Yamamoto T, Shoji J, Arimura T, Nagano E, Tsuruoka H, Sato K (2010). An empirical research on parental support in child guidance center (4) an empirical research of effectiveness, validity, assessment and adaptation of parental support (in Japanese). Reports of Studies of Japan Child and Family Research Institute.

